# Drug-Drug Interactions among Patients Hospitalized with COVID-19 in Greece

**DOI:** 10.3390/jcm11237172

**Published:** 2022-12-02

**Authors:** Marios Spanakis, Petros Ioannou, Sotiris Tzalis, Vasiliki Papakosta, Evridiki Patelarou, Nikos Tzanakis, Athina Patelarou, Diamantis P. Kofteridis

**Affiliations:** 1 Department of Nursing, School of Health Sciences, Hellenic Mediterranean University, 71004 Heraklion, Greece; 2 Computational Biomedicine Laboratory, Institute of Computer Science, Foundation for Research and Technology-Hellas (FORTH), 70013 Heraklion, Greece; 3 Department of Internal Medicine and Infectious Diseases, University Hospital of Heraklion, 71110 Heraklion, Greece; 4 Department of Respiratory Medicine, University Hospital of Heraklion, Medical School, University of Crete, 71003 Heraklion, Greece

**Keywords:** COVID-19, drug-drug interactions, SARS-CoV-2, pharmacotherapy, adverse drug reactions

## Abstract

The modulation of the pharmacological action of drugs due to drug-drug interactions (DDIs) is a critical issue in healthcare. The aim of this study was to evaluate the prevalence and the clinical significance of potential DDIs in patients admitted to the University Hospital of Heraklion in Greece with coronavirus disease 2019 (COVID-19). Cardiovascular disorders (58.4%) and diabetes (types I and II) (29.6%) were the most common comorbidities. A high occurrence of DDIs was observed, and clinically significant DDIs that may hamper response to treatment represented 40.3% of cases on admission, 21% during hospitalization, and 40.7% upon discharge. Polypharmacy and comorbidities were associated with a higher prevalence of DDIs in a statistically significant way (*p* < 0.05, 95% CI). Clinically significant DDIs and increased C-reactive protein values upon admission were associated with prolonged hospitalization. The results reveal that patients admitted due to COVID-19 in Greece often have an additional burden of DDIs that healthcare teams should approach and resolve.

## 1. Introduction

The severe acute respiratory syndrome coronavirus 2 (SARS-CoV-2) that causes the coronavirus disease (COVID-19) has infected more than 610 million people worldwide and is responsible, until today, for more than 6.5 million deaths since its outbreak in China in 2019 [1,2]. Although it is expected that the accumulation of mutations and the currently emerging SARS-CoV-2 variants will likely lead to reduced mortality rates and transform the pandemic into its endemic phase, COVID-19 is still pressing the healthcare systems [3,4,5,6]. The most frequently associated factors for severe illness and hospitalization from COVID-19 are cardiovascular diseases, obesity, dyslipidemias, diabetes, respiratory diseases, and cancer along with aging and lack of vaccination [7,8]. Generally, people with more than one underlying health condition are at increased risk for hospitalization and acute disease [9,10,11]. Hence, COVID-19 is a common underlying condition for so many different clinical cases of patients with comorbidities [12,13]. As medical teams in COVID-19 wards try to follow efficient medication protocols for optimal healthcare provision for COVID-19 hospitalized patients, they should also adjust to each patient’s needs in order to avoid clinically significant drug-drug interactions (DDIs) from co-medications that can cause complications, adverse drug reactions (ADRs), and prolong hospitalization [14].

DDIs refer to modulations of the pharmacological profile of a drug from co-administered ones. These alterations can be related to pharmacokinetic (PK) processes of absorption, distribution, metabolism, and elimination (ADME), such as modulation of metabolic enzymes (i.e., cytochrome P450, CYPs) or transporter proteins (P-glycoprotein, P-gp; organic anion transporters, OATP) and plasma proteins (i.e., albumin). DDIs can also be related to pharmacodynamic mechanisms in the site of action and/or in other tissues [15]. Clinically significant DDIs may result in ADRs and side effects that further impair patients’ health, obscuring the treatment outcome and prolonging hospitalization [16,17]. In the case of COVID-19, the scientific community gave an early warning of the risk for DDIs from the several applied protocols [14,18,19,20,21,22,23,24]. Even with the introduction of nirmatrelvir/ritonavir (Paxlovid^TM^), there were regulatory check lists to assist clinicians in evaluating potential DDIs and other patient factors prior to any administration [25]. In addition, for recently approved monoclonal antibodies such as casirivimab/imdevimab (REGEN-COV) and sotrovimab (Xevudy), no official DDI studies have been performed [26,27]. They are not renally eliminated or metabolized by CYP enzymes; hence, DDIs are unlikely with drugs that are substrates, inducers, or inhibitors of CYP enzymes or excreted through the kidneys. However, caution is advised, and healthcare providers should be aware of this new field of DDIs in COVID-19 if any observation should be reported, (e.g., interactions with COVID-19 vaccinations) in terms of pharmacovigilance. In a recent review, we described the risk for potential DDIs with drugs introduced for COVID-19 for patients with respiratory disorders and presented underlying pharmacological mechanisms, their significance, and possible clinical symptoms that could be recognized by healthcare teams staffing the COVID-19 wards [18].

Advancing our approaches, the aim of this work was to record and analyze the occurrence of DDIs in COVID-19 patients hospitalized over the previous months in the University Hospital of Heraklion in Greece. The study analyzes the prevalence of DDIs among the medications administered to those patients, their clinical significance, and their potential impact on hospitalization.

## 2. Materials and Methods

### 2.1. Study Design and Ethics Approval

This observational, single-center descriptive study was conducted over a 6-month period (January–June 2022) in the COVID-19 department of the University Hospital of Heraklion in Crete, Greece. The study followed the rules of the Declaration of Helsinki of 1975, as revised in 2013, and it complied with the General Data Protection Regulation (GDPR). It was approved by both the Hellenic Mediterranean University (51/4 March 2021) and the University Hospital’s ethics committee (16105/13 October 2021). The study follows guidelines (Table 1) for reporting observational studies (Strengthening the Reporting of Observational Studies in Epidemiology—STROBE) [28].

Patients who were admitted to the COVID-19 ward, had laboratory confirmation of SARS-CoV-2 infection through reverse transcription polymerase chain reaction (RT-PCR), and willingly signed the informed consent form were analyzed. All participants were adults (18–65 years old) or elders (>65 years old). Participation was based on free will. Patients who did not understand the terms of participation and consent were excluded from the study. Patients who were not hospitalized were excluded. All data were collected and analyzed anonymously, and no interventions were made regarding healthcare provision during hospitalization.

Patients’ data were collected from the hospital’s electronic medical record system and included demographics, clinical, and laboratory data, as well as medication regimens upon admission, during hospitalization, and on the day of discharge. Polypharmacy was classified as co-administration of five or more (≥5) medications. If a medication contained more than one pharmacologically active compound, they were considered different medications (i.e., ipratropium with albuterol). Medications were classified according to the Anatomical Therapeutic Chemical (ATC) classification system (Appendix A) and were presented with its second level anatomical group-therapeutic subgroup, i.e., ATC-X00.

### 2.2. Evaluation of DDIs

The analysis of the DDIs and their clinical significance have been previously presented [29,30]. Briefly, medication regimens were recorded and DDIs were detected using available online drug interaction checker tools (Medscape and Drugs.com; accessed on 1 April till 30 July 2022), considering the level of evidence available in the literature describing the significance, such as experts’ opinions, in silico, in vitro, in vivo data, clinical studies, reviews, the Summary of Product Characteristics (SmPC), and reports from regulatory authorities. The DDIs were characterized as pharmacokinetic (PK) or pharmacodynamic (PD) and as “Serious-Use alternative” “Use with caution-Monitor” and “Moderate-Minor” DDIs. The ATC groups that are paired in recorded DDIs were represented with circos diagrams that were generated with Circos Table Viewer v0.63-9© (http://circos.ca, accessed on September 2022) [31].

### 2.3. Statistical Analysis

Data are presented as numbers or percentages for continuous variables and expressed as mean values ± standard deviation (±SD). Polypharmacy and clinically significant DDIs that were recorded were evaluated as factors that prolong hospitalization stay. In this respect, a Mann—Whitney *t*-test was performed with 95% confidence intervals (CI) and a statistical significance of *p* < 0.05 using GraphPad Prism version 8.0.1 for Windows, GraphPad Software, San Diego, CA, USA, www.graphpad.com.

## 3. Results

### 3.1. Patient Demographics, Comorbidities, and Clinical Status

**Figure 1:**
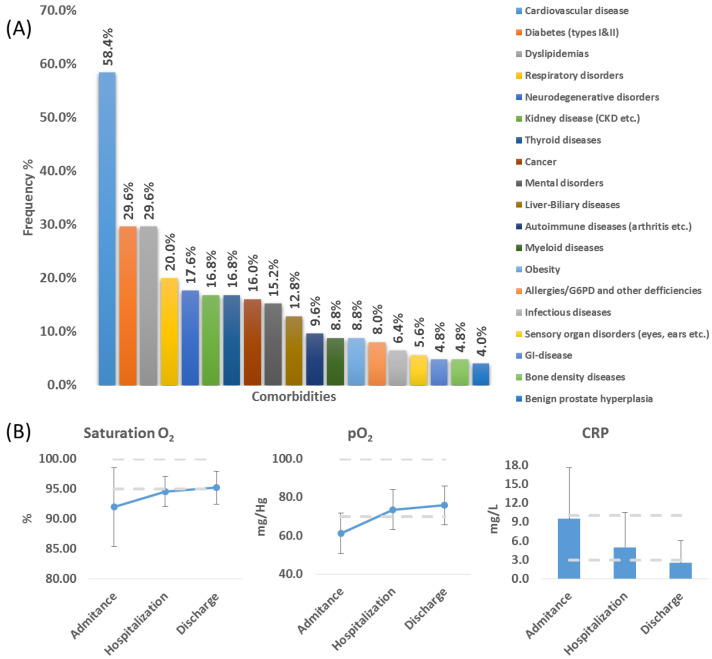
(**A**) Comorbidities that were recorded among the cohort of COVID-19 patients. (**B**) Clinical data of oxygen saturation (saturation O_2_), partial pressure of O_2_ (pO_2_), and C-reactive protein (CRP) for those patients. The gray dashed lines represent the normal laboratory values.

The study enrolled 125 patients (76 males and 49 females) who had a positive laboratory test for SARS-CoV-2 infection (RT-PCR confirmed) and signed the informed consent form. Their demographic characteristics are presented in Table 2. They were residents of urban areas (52%), their mean age was 72.5 (±14.7) years old, with a BMI of 33.1 (±9.12) and 30% of them were smokers (Table 2). From the 125 patients, 52% were fully vaccinated (3 doses of Comirnaty, Spikevax, or 2 doses of Vaxzervria with a 3rd dose of Comirnaty), 15% were partially vaccinated (1 or 2 doses of the aforementioned vaccines), and 23% had not received any vaccine. Regarding comorbidities, a mean number of four comorbidities per patient was recorded, while 8 patients had no other medical conditions. Cardiovascular disorders (58.4%), diabetes (types I and II) (29.6%), dyslipidemias (29.6%), and respiratory disorders (20%) were the most often recorded comorbidities (Figure 1A). As far as their clinical status, upon admission, patients had low saturation (60%) and partial pressure of oxygen (O_2_) (63.2%), as well as high C-reactive protein (CRP) values (34.4%) (Figure 1B). The full clinical dataset as recorded is available in the Supplementary Materials. Their median hospitalization duration was 7 days (mean 8.6). The mortality within the study group was 13%.

### 3.2. Drugs Administered to COVID-19 Patients

**Figure 2:**
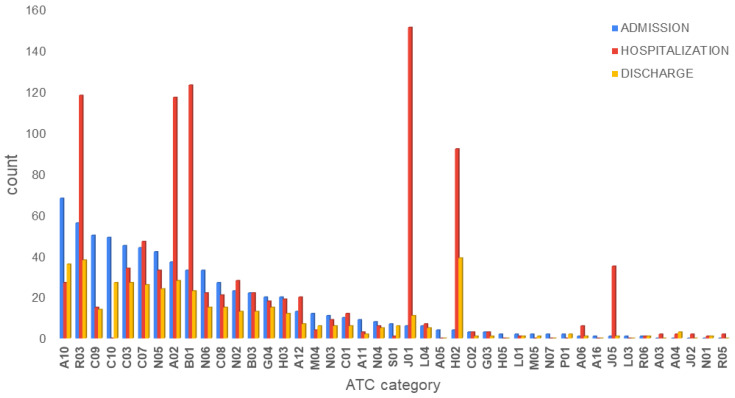
ATC drug categories that were administered to COVID-19 patients during admission (blue bars), hospitalization (red bars), and discharge (yellow bars).

Patients were prescribed cardiovascular drugs (ATC-C01, C03, C07, C08, and C09), antidiabetic drugs (ATC-A10), and lipid-modifying agents (ATC-C10), along with drugs for benign prostatic hyperplasia (ATC-G04), respiratory disorders (ATC-R03), antipsychotics (ATC-N05), and antidepressants (ATC-N06). These categories were also the most prevalent upon discharge. During hospitalization, patients’ medications were revised and modified. The general approach involved the cessation of antihypertensives, antidiabetic drugs, lipid-modifying agents, and per os anticoagulants and the initiation of therapy with low molecular weight heparin (LMWH), dexamethasone, and remdesivir. In patients with respiratory infections, cephalosporins (3rd generation) were added with or without macrolide antibiotics, and less often quinolones. To provide gastrointestinal (GI) protection, esomeprazole, or other proton-pump inhibitors (PPIs) were used. Overall, 175 different medications were recorded upon admission, which were reduced to 149 during hospitalization and 144 upon discharge. Polypharmacy (≥5 medications) accounted for 55.5% upon admission, 82% during hospitalization, and 55.6% upon discharge. An average number (±interquartile range) of 6 (±4) drugs per patient were recorded upon admission, 8 (±6) during hospitalization, and 6 (±4) upon discharge. Fitting the second level of the ATC index, they were organized into 43 different ATC groups and presented in Figure 2. During hospitalization, a rise was observed in the administration of antithrombotic agents (ATC-B01), antibacterials (ATC-J01), and antivirals (ATC-J05) for systemic use; drugs for obstructive airway diseases (ATC-R03); corticosteroids for systemic use (ATC-H02); and PPIs (ATC-A02). Corticosteroids for systemic use (ATC-H02) and drugs for obstructive airway diseases (ATC-R03) were also continued upon discharge. The most often administered drugs during hospitalization were esomeprazole (89.6%), enoxaparin (80.8%), dexamethasone (51.2%), ipratropium (32.8%), ceftriaxone (26.4%), remdesivir (22.4%), budesonide (21.6%), furosemide (18.4%), methylprednisolone (16.8%), aspirin (16%), insulin (16%), and azithromycin (15.2%).

### 3.3. DDIs, Clinical Significance, and Related Pharmacological Mechanisms

**Figure 3:**
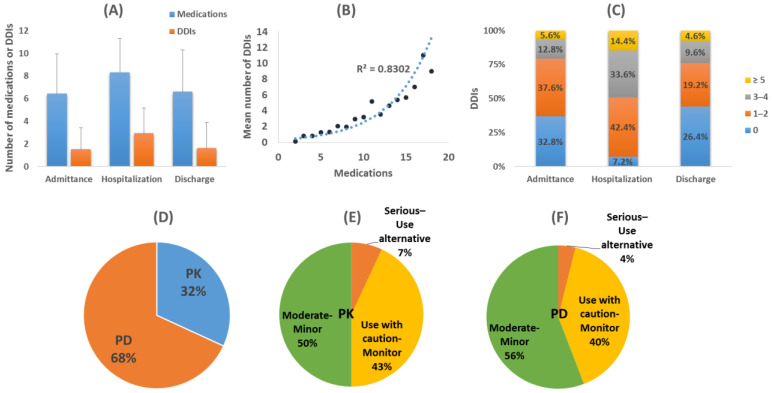
(**A**) Average drugs administered and drug-drug interactions (DDIs) per each time point. (**B**) Average number of DDIs over number of administered drugs. (**C**) Patient exposure in DDIs (%) for each time point. (**D**) Pharmacological mechanisms and clinical significance for (**E**) Pharmacokinetic (PK)-DDIs and (**F**) Pharmacodynamic (PD)-DDIs.

Drug pairs related to potential DDIs were found in 67.2% of patients during admission, 92.8% during hospitalization, and 60% upon discharge. Overall, 572 cases from 226 different drug combinations were recorded as potential DDIs. There was an average value of 3 DDIs (min = 0, max = 13) during hospitalization, whereas 2 DDIs (min = 0, max = 12) were the mean recorded value during admission and discharge (Figure 3A). There was an exponential correlation (Rc^2^ = 0.902) between the average number of DDIs and the number of medications administered (Figure 3B). During hospitalization, patients were exposed to a higher number of DDIs than during admission or discharge (Figure 3C).

Clinically significant DDIs such as “Serious-Use alternative” and “Use with caution-Monitor) represented 40.3% of cases at admission, 21% during hospitalization, and 40.2% upon discharge. “Moderate-Minor” DDIs were 59.8% upon admission, 77.6% during hospitalization, and 59.8% upon discharge. The analysis of DDIs revealed that 32% of them were based on PK mechanisms and the rest, 68%, on PD ones (Figure 3D). For PK-DDIs, 7% of them were recognized as “Serious-Use alternative” and 43% as “Use with caution-Monitor” and for PD-DDIs, 4% were recognized as “Serious-Use alternative” and 40% as “Use with caution-Monitor” (Figure 3E,F).

**Figure 4:**
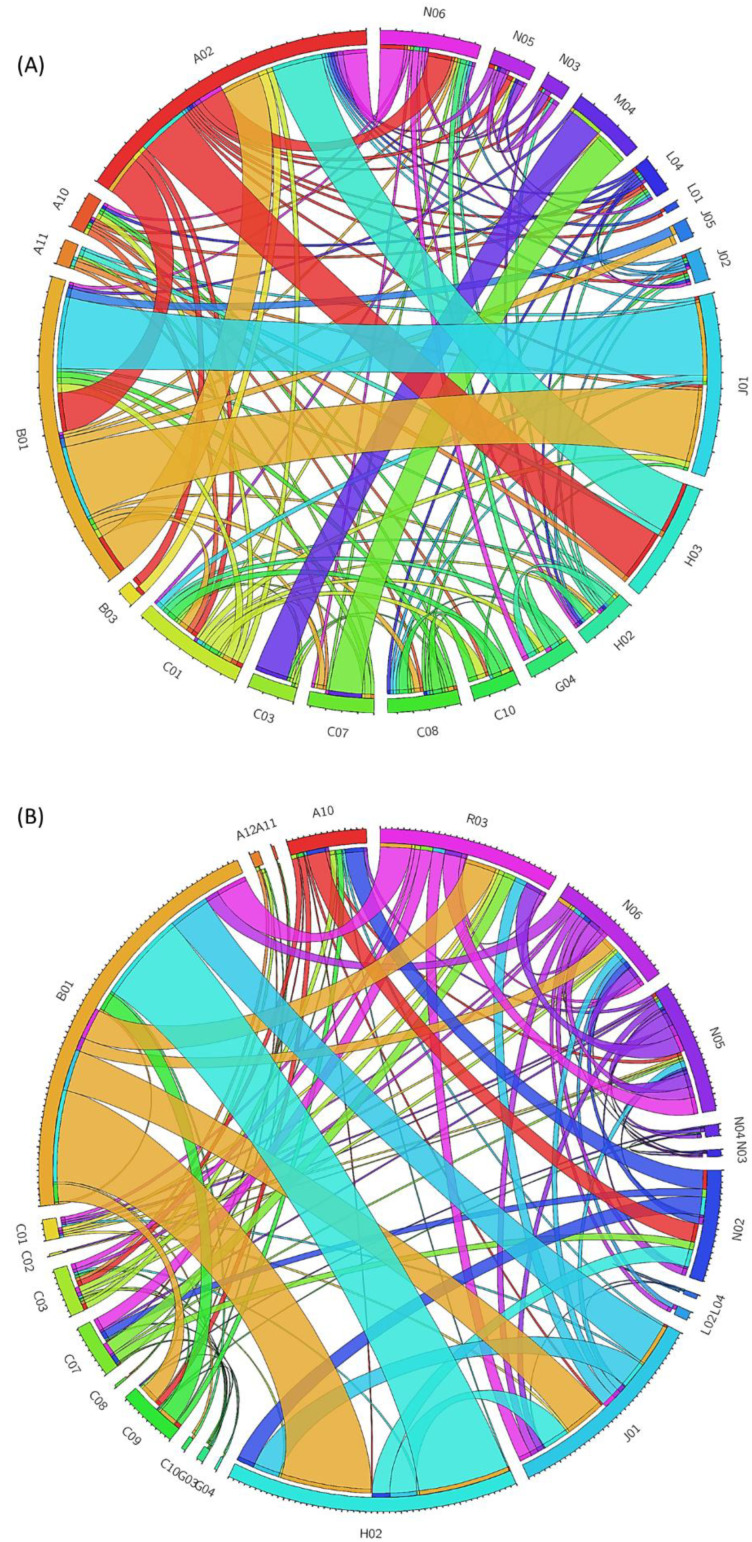
Circos plots displaying the interconnections between ATC categories that can lead to (**A**) pharmacokinetic drug-drug interactions and (**B**) pharmacodynamic drug-drug interactions as described in this study.

PK-DDIs were related to pharmacological mechanisms involving inhibition or induction of CYP- mediated metabolism, P-gp or OATP-mediated transport, modulation of GI absorption due to alteration of pH-dependent solubility, protein binding competition, and modulation of renal elimination that could lead to elevated concentrations for one drug and prolonged action (Table 3). PD-DDIs were related to cases that required INR monitoring, QT prolongation, imbalances in electrolytes (i.e., potassium and sodium) and blood glucose, drug-induced injuries, deterioration in renal function, and GI side effects (Table 3). Examples of drug pairs that lead to PK or PD DDIs are presented in Table 4. The overall ATC categories that were found to pair for DDIs are represented with the circos diagrams in Figure 4.

The circos diagram for PK (Figure 4A) shows an interconnection in DDIs between the use of antithrombotic agents (ATC-B01) and antibiotics (ATC-J01) (i.e., acenocoumarol and ceftriaxone) or antivirals (ATC-J05) (i.e., acenocoumarol and remdesivir) as well as with PPIs (ATC-A02) (i.e., clopidogrel-esomeprazole). The impact of PPIs on CYPs is also related to PK-DDIs with central nervous system drugs such as ATC-N06 (i.e., omeprazole and escitalopram). The modulation of CYPs and transporters can also be related to PK-DDIs such as in cases of cardiovascular medications (ATC-C01, C07, C08, and C10) (i.e., ranolazine with statins). The use of corticosteroids can impact the PK profiles of alpha-adrenoreceptor antagonists (ATC-G04) due to CYP induction. Finally, the co-administration of azithromycin (ATC-J01) can modulate the PK profile of statins (ATC-C10). Regarding circos of PD-DDIs (Figure 4B), the potential for DDIs of antithrombotic agents (ATC-B01), corticosteroids (ATC-H02), drugs for obstructive airway diseases (ATC-R03), and psychoanaleptics (ATC-N06) was observed, all of which can modulate the anticoagulation action and require INR monitoring, whereas the co-administration of agents acting on the renin–angiotensin system (ATC-C09) can cause hyperkaliemia. The use of antibacterials for systemic use (ATC-J01) can also modulate the INR values (i.e., enoxaparin and ceftaroline), whereas quinolones with corticosteroids (ATC-H02) can lead to tendon rupture and muscle damage. The co-administration of drugs for obstructive airway diseases (ATC-R03), especially β2-agonists, with cardiovascular medications such as β-blockers (ATC-C07), can result in acute bronchospasm, whereas co-administration of anticholinergics with CNS drugs (ATC-N05), such as olanzapine, can result in additive anticholinergic effects and possible hypoglycemia [32]. There are also PD-DDIs between NSAIDs and aspirin (ATC-N02), with angiotensin receptor blockers or angiotensin converting enzyme inhibitors (ATC-C09), as well as with anti-diabetic medications (ATC-A10). For CNS drugs (ATC-N03, N04, N05, and N06), there is always the risk of PD-synergism that can cause respiratory depression (i.e., carbamazepine with levomepromazine, biperiden) and additive anticholinergic effects (i.e., donepezil, haloperidol), whereas the co-administration with β-blockers can cause hypotensive effects (i.e., atenolol, zolpidem). The administration of immunosuppressants (ATC-L04) can result in DDIs either due to synergisms and the risk of serious infection (i.e., abatacept and adalimumab) or impact the CYP abundance and change drugs’ intrinsic clearance (PK-DDIs) through the liver (i.e., anakinra and alprazolam). Finally, PD-DDIs that result in QT prolongation can occur with the co-administration of antiarrhythmics (ATC-C01), antibacterials (ATC-J01), CNS medications (ATC-N05, ATC-N06), and drugs for obstructive airway diseases (ATC-R03).

### 3.4. Impact of Polypharmacy and DDIs on the Hospitalization Status of COVID-19 Patients

**Figure 5:**
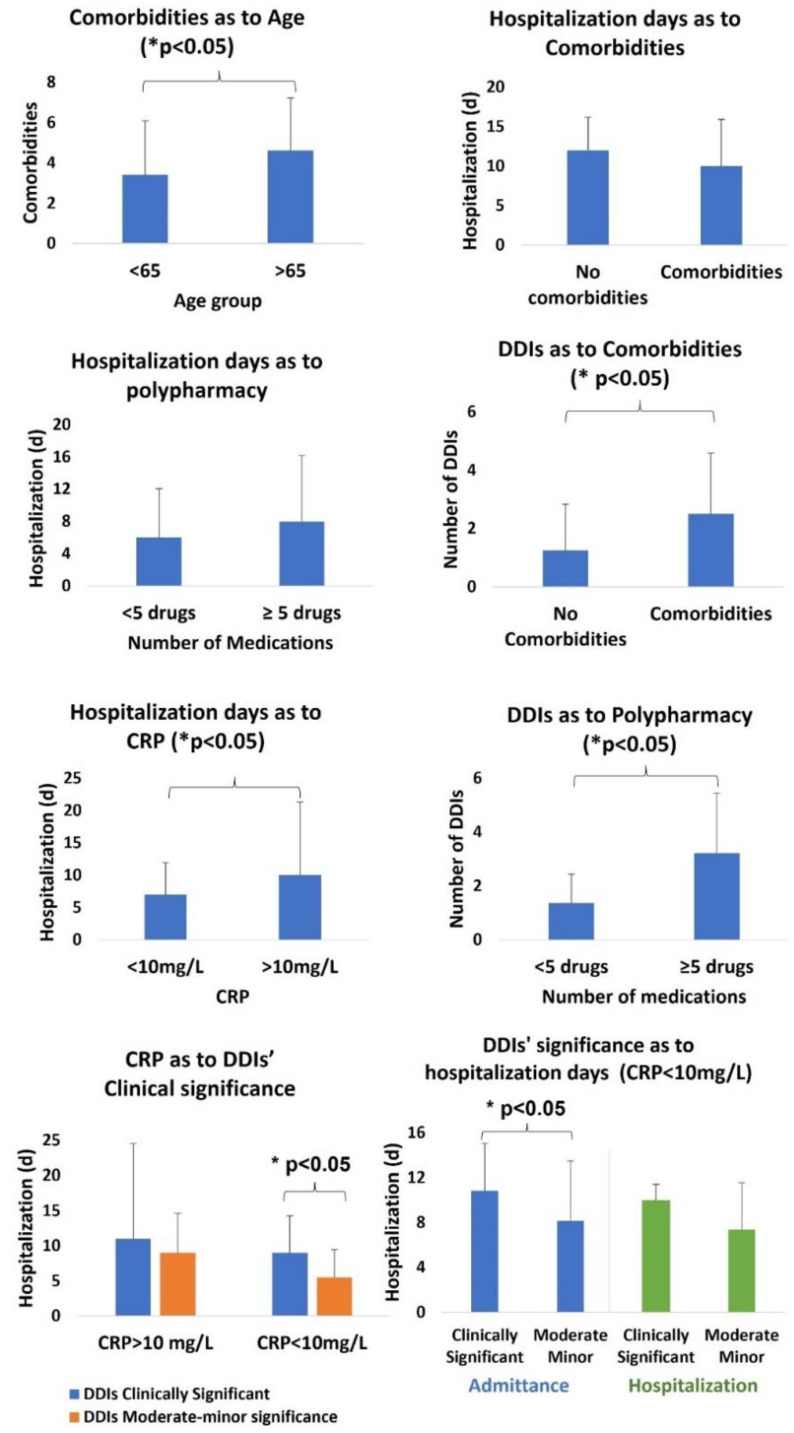
Differences in hospitalization days for COVID-19 patients due to comorbidities, age, polypharmacy, DDIs, and CRP values. Statistically significant differences (* *p* < 0.05) are noted with horizontal brackets.

Patients over 65 had a higher number of comorbidities than adult patients below 65 (*p* < 0.05, 95% CI), although age and comorbidities did not seem to play any role regarding hospitalization days (*p* > 0.05, 95% CI). High CRP values (>10 mg/L) upon admission were associated with prolonged hospitalization days (*p* < 0.05, 95% CI). Polypharmacy was not associated with prolonged hospitalization (*p* > 0.05, 95% CI). However, patients with comorbidities had an increased number of DDIs (*p* < 0.05, 95% CI), and their prevalence was higher in a statistically significant way for patients in a polypharmacy state, independent of the time point (admission, hospitalization, and discharge) (*p* < 0.05, 95% CI). As to the clinical significance of DDIs, an apparent association between the detection of clinically significant interactions and longer hospital days was recorded. The association was mainly found in patients with CRP < 10 mg/L and clinically significant DDIs in their medication regimens upon admission (*p* < 0.05, 95%CI) (Figure 5).

## 4. Discussion

Healthcare provision for patients with SARS-CoV-2 infection has proven to be a very complicated issue that will bring transformative changes in how healthcare is provided, especially in intensive care units [33,34]. The pervasiveness of the disease creates numerous and complicated clinical scenarios for COVID-19 patients with chronic diseases and complex therapeutic schemes, which are linked with an increased risk of adverse clinical outcomes [35,36]. One of the main risk factors for ADRs is clinically significant DDIs. This work presented the results of an observational study regarding the prevalence of DDIs among 125 patients hospitalized in the COVID-19 department of the University Hospital of Heraklion in Greece. Comparably to previously published works regarding the clinical characteristics of COVID-19 patients, the patients in this study had an average number of four comorbidities: cardiovascular disease (i.e., coronary artery disease, cardiomyopathies, hypertension, etc.); diabetes (types I and II); obesity; dyslipidemias; respiratory disorders; CNS disorders; cancer (occurred most often) (Figure 1) [12,37,38,39]. Furthermore, an additional factor related to prolonged hospitalization was the increased CRP values (34.4% of the cases). CRP has also been proposed as a prognostic indicator for the assessment of disease severity in COVID-19 [40].

Potentially interacting drug pairs were found in 67.2% of patients during admission, 92.8% during hospitalization, and 60% upon discharge. Hospitalization’s high prevalence of interacting drug pairs is mostly due to the co-administration of LMWH with corticosteroids (i.e., dexamethasone), which may increase INR, a potential DDI of moderate significance. Nevertheless, the use of LMHW for anticoagulation and alleviation of inflammation mechanisms, along with dexamethasone’s effect in reducing ARDs risk, represents a prominent treatment for COVID-19 worldwide, endorsed by many medical societies’ guidelines and with a lot of evidence to support it [41,42,43,44]. Thus, careful administration of these medications should be performed while taking into consideration the underlying medical conditions, COVID-19 disease severity, as well as dexamethasone’s side effects of hyperglycemia, hypernatremia, hypertension, and potentiation of anticoagulant effects [41,45].

Pharmacological mechanisms of DDIs were related to pharmacokinetic processes in 32% of cases, and the rest (68% of cases) were related to pharmacodynamic pathways. PK-DDIs were mostly related to inhibition of CYP-mediated metabolism from perpetrator drugs, which may alter patients’ systemic drug concentrations, thus modulating its pharmacological action (Table 3 and Table 4). For example, inhibition of clopidogrel’s CYP2C19-mediated metabolism by PPIs (e.g., esomeprazole, omeprazole) may result in reduced concentrations of clopidogrel’s active metabolite and reduced antiplatelet action, whereas the co-administration of escitalopram and PPIs may lead to elevated concentrations and thus enhanced escitalopram’s pharmacological action [46,47]. PD-DDIs were mostly associated with synergistic effects of drugs that may potentiate pharmacological outcomes or increase the risk for side effects (i.e., potentiation of the anticoagulation action of antithrombotic agents or drugs that contribute to QT prolongation) [45,48].

Clinically significant DDIs of “Serious-Use alternative” or “Use with caution-Monitor” management were found in 40.3% of cases upon admission, 21% during hospitalization, and 40.7% upon discharge. The increased number of clinically significant DDIs upon admission is in line with previous observations regarding polypharmacy and the occurrence of DDIs among outpatient prescriptions in Greece [49,50]. Clinically significant DDIs at the time of admission were also associated with a longer hospital stay (*p* < 0.05, 95% CI). In addition, they were mostly related to PK-DDIs since the “Serious-Use alternative” DDIs represent 7% of PK-DDIs versus 4% of PD-DDIs. This is also attributed to the fact that the evaluation of significance in cases of PK-DDIs is more feasible compared to PD-DDIs since they have specific endpoints and obvious mechanisms, whereas PD-DDIs have varied outcomes, more complicated mechanisms, and clinical effects [48]. On the other hand, a reduction in the clinical significance of DDIs during hospitalization was observed compared to admission. This can be attributed to the fact that specialized and multidisciplinary healthcare teams in medical wards have additional clinical information such as laboratory values and a full medication list, which allows them to be more compliant with evidence-based clinical guidelines and proceed to a better risk/benefit analysis with fewer medication errors [29,51,52,53].

The occurrence of DDIs was higher for COVID-19 patients in a polypharmacy state (≥5 drugs). Polypharmacy is a documented risk factor for adverse drug reactions from clinically significant DDIs, especially in clinically ill patients [29,30,54]. Regarding COVID-19, polypharmacy and comorbidities have been described as risk factors for clinically significant DDIs early in the first wave, with the main concern being drug combinations that increase the risk for QT prolongation [14,35,55]. Previous studies have shown an increased prevalence of DDIs in COVD-19 patients undergoing treatment with lopinavir or ritonavir, and recognized risk factors include polypharmacy, age over 65, respiratory or CNS disorders, and dyslipidemias [56]. In our work, lopinavir and ritonavir were not administered according to the medical protocols that were followed. However, risk factors such as polypharmacy and comorbidities were also correlated with the high prevalence of DDIs (Figure 5). In addition, QT prolongation (Table 3) was the second most common potential outcome among the PD-DDIs due to the co-administration of antiarrhythmics (ATC-C01), antibacterials (ATC-J01), and especially azithromycin, CNS medications (ATC-N05, ATC-N06), and drugs for obstructive airway diseases (ATC-R03). In other studies, hypoglycemia and QT prolongation have been reported to be the most common predicted outcomes of DDIs, with risk factors being polypharmacy and comorbidities, whereas a correlation between the average number of drugs and the number of medications was observed [57]. In our study, the risk for hypoglycemia was less frequent (Table 3), but again, we observed an association between the number of drugs and DDIs (Figure 5). Overall, treating physicians in COVID-19 wards should be aware of potential PK-DDIs when antithrombotic agents (ATC-B01), antibiotics (ATC-J01), and antivirals (ATC-J05), as well as PPIs (ATC-A02), are co-administered along with cardiovascular medications (ATC-C). Regarding PD-DDIs, they should consider potential DDIs if arrhythmias occur due to the co-administration of drugs that prolong the QT interval. In addition, if INR values are modulated, it can be related to the co-administration of antithrombotic agents (ATC-B01), antibacterial agents (ATC-J01), corticosteroids (ATC-H02), drugs for obstructive airway diseases (ATC-R03), and psychoanaleptics (ATC-N06). They should also be aware of potential synergistic PD-DDIs that can be associated with the co-administration of CNS drugs (ATC-N) (Table 4 and Figure 4) [14,21,35,55,58,59].

Until today, several risk score calculators have been developed to aid clinical decisions for COVID-19 patients [60,61]. Usually, these approaches take into consideration the clinical characteristics and risk factors that, until today, have been recognized for COVID-19. These patients are usually under polypharmacy conditions and thus at risk for ADRs from DDIs. This is a factor that should always be considered and incorporated into risk score calculators. Previous research has demonstrated that COVID-19 patients have a high prevalence of DDIs, especially those on polypharmacy [57,62]. This was evident also in this study, which estimated an exponential correlation between the number of medications and the average number of DDIs. On the subject of the apparent association between clinically significant DDIs and prolonged hospitalization, the current study cannot provide a causality conclusion as to whether DDIs actually prolong hospitalization or occur due to complex treatment regimens in prolonged hospitalized patients. Hence, prospective studies are needed to further clarify the possible causality of the observation for COVID-19 patients [16,17,63]. On the other hand, the reduction in the clinical significance of DDIs reveals that specialized medical teams can reduce the burden of DDIs among hospitalized patients and improve the healthcare provided. Overall, clinical signs, such as excessive or reduced drug action, modulation of INR, QT prolongation, and Torsades de Pointes (TdP), changes in electrolytes (K^+^, Na^+^), hyper/hypoglycemia, sedation, respiratory depression, and muscle, kidney, or liver dysfunctions, should be approached from a DDIs point of view for hospitalized COVID-19 patients, similar to those described in other works and in this study.

Limitations of the study that can be mentioned are the relatively condensed sample size and that it took place in one hospital. However, the analysis of the data revealed a good correlation between the clinical characteristics of this cohort and the occurrence of DDIs compared with the literature for COVID-19 [64].

## 5. Conclusions

The current study examined the prevalence of DDIs among patients in the COVID-19 department of the University Hospital of Heraklion in Greece. Clinically significant DDIs related to comorbidities and polypharmacy that can hinder treatment response and complicate hospitalization were recorded mostly upon admission and to a lesser extent during hospitalization. COVID-19, even as an endemic disease, will remain a permanent burden that challenges healthcare provision. Clinicians should be aware of and follow stepwise approaches to control inflammation and prevent acute respiratory distress syndrome (ARDS), as well as to avoid or minimize clinically significant DDIs and relative ADRs that may complicate hospitalization and treatment outcomes.

## Figures and Tables

**Table 1:** STROBE information for the study regarding methods and results.

Methods	
Study design	Observational, retrospective, and descriptive study of DDIs in patients admitted with COVID-19
Setting	COVID-19 ward, University Hospital Heraklion, Crete, Greece
Participants	Patients requiring inpatient treatment for COVID-19
Variables	Record of demographic characteristics; clinical values; comorbidities; medication regimens; number of DDIs; clinical significance; hospitalization days
Data sources/ measurement	DDIs are based on literature searches and relative databases (Medscape, drugs.com, accessed on 1 April–30 July 2022)
Study size	Target population: patients admitted with COVID-19 Study population: signed informed consent form
Bias	Diligence in informing the purpose and objectives of the study Diligence in recording the medication regimens in the correct time periods Recording demographics and medication regimens Analysis of data regarding the significance
**Results**	
Participants	The informed consent form was signed by 125 participants (76 males/49 females)
Descriptive data	Average comorbidities: 4.0 Average hospitalization days: 8.6 (median 7) Admittance: laboratory confirmation for SARS-CoV-2 Vaccination status: 52% complete, 15% partial, and 23% none Mortality: 13%
Outcome data	Comorbidities: Cardiovascular disorders (58.4%) and diabetes (types I and II) (29.6%) 226 unique DDIs PK-DDIs: 32.0% and PD-DDIs: 68.0%
Main results	Patients with at least 1 potential DDI: 67.2% (admission), 92.8% (hospitalization), and 60% (discharge) Clinically significant DDIs: 40.3% (admission), 21% (hospitalization), and 40.7% (discharge) Patients with comorbidities had an increased number of DDIs (*p* < 0.05, 95% CI) DDIs were more prevalent for patients in a polypharmacy state (*p* < 0.05, 95% CI) Exponential correlation between DDIs and number of drugs Clinically significant DDIs were observed in patients that also had prolonged hospitalization (*p* < 0.05, 95% CI)

COVID-19: coronavirus disease; DDIs: drug-drug interactions; SARS-CoV-2: severe acute respiratory syndrome coronavirus 2; PK: pharmacokinetic; PD: pharmacodynamic.

**Table 2:** Demographic characteristics of patients enrolled in the study.

Demographics	Mean (±Standard Deviation)	Min/Max
Age (y)	72.5 (±14.7)	33/97
Height (m)	1.7 (±0.2)	1.2/1.9
Weight (kg)	81.3 (±19.2)	48.0/130.0
Body Mass Index (BMI, kg/m^2^)	33.1 (±9.1)	22.2/50.0
Comorbidities	4 (±3)	0/12
Vaccination	5% (3 doses); 14.4% (2 doses); 81.4% (1 or no dose)	
Duration of hospitalization (d)	8.6 (±4.7) (median = 7)	2/74
Mortality	13% (13% fully; 1% partially and 20% unvaccinated)	
Polypharmacy (≥5 drugs)	Admission = 55.2%; Hospitalization = 82.0%; Discharge = 55.8%	
**Residence & Social Habits**		
Urban	65 (52%)	
Suburban	9 (7%)	
Semi-urban	14 (11%)	
Rural	35 (28%)	
Smoking	38 (30%)	

**Table 3:** Pharmacological mechanisms of the recorded DDIs and their frequency (N).

Mechanisms of PK-DDIs	N
**Inhibition of CYP-mediated metabolism**	39
**Reduced bioavailability due to pH-dependent solubility**	23
**Reduced metabolism** (non-CYP)	21
Dual inhibition **of CYP metabolism, P-gp, or other proteins transport**	13
**Increased serum urate and Ct of metabolite (oxypurinol)**	11
**Inhibition of P-gp-mediated transport**	10
**Induction of CYP-mediated metabolism**	10
**Modulation of GI absorption**	4
Inhibition of influx-**mediated transport (e.g., OAT1B1 or OCT2)**	3
**Renal tubular clearance**	2
**Protein binding competition**	2
Dual induction **of CYP-mediated metabolism and P-gp transport**	2
**Restore suppressed CYP expression caused by inflammation**	1
**Decrease tubular secretion**	1
**Mechanisms of PD-DDIs**	
**Modulation of anticoagulation action and altered INR-monitor**	183
**QT prolongation**	63
**Risk for hyperkalemia**	25
**Risk of tendon rupture**	24
**Risk for hypoglycemia**	18
**GI side effects**	16
**Deterioration in renal function (elderly)**	11
**PD antagonism-acute bronchospasm**	11
**PD synergism, sedation, and respiratory depression**	9
**PD antagonism-altered antihypertensive response**	7
**Risk for hypokalemia**	7
**Risk for hyperglycemia**	6
**Reduce renal function and antihypertensive effect of ACE inhibitors**	4
**Risk for serotonin syndrome**	4
**PD synergism-hypotensive effects**	4
**Risk for hyponatremia**	3
**Additive anticholinergic effects**	3
**PD-antagonism decreased effect of levodopa**	2
**Hypotension with hyperglycemia**	2
**PD-synergism cardiovascular side effects**	2
**Quinolone administration may result in hyper- or hypoglycemia**	2
**Risk for nephrotoxicity and/or ototoxicity.**	1
**PD-antagonism of Ca^2+^ with Ca^2+^ channel blockers**	1
**PD-synergism and excessive parasympatholytic effects**	1
**PD-synergism increased risk for serious infection**	1

PK: pharmacokinetic; PD: pharmacodynamic; DDI: drug-drug interactions; CYP: cytochrome P450; OCT: organic cation transporter; GI: gastrointestinal tract; OATP1B1: organic anion transporter 1B1; QT: interval between the heart’s contraction and relaxation; ACE: angiotensin-converting enzyme.

**Table 4:** Examples of drug pairs that can lead to DDIs as recorded in this study (bold are marked drugs used in COVID-19 medication protocols). (Abbreviations: ATC: anatomical therapeutic classification index, SUA: Serious-Use alternative, Monitor: use with caution-monitor, Moderate: moderate-minor, N: number of cases).

Drug A	Drug B	ATC		Pharmacological Outcome	Significance	N
**Acenocoumarol**	**Methylprednisolone**	B01	H02	PD-INR-monitor	Monitor	3
	Remdesivir		J05		Monitor	3
	Ceftriaxone		J01		SUA	2
	Rosuvastatin		A02		Moderate	2
	Esomeprazole		C10	PK-CYP inhibition	Moderate	3
**Allopurinol**	Furosemide	M04	C03	PK-Ct metabolite	Monitor	11
**Amiodarone**	Metformin	C01	A10	PK-renal clearance	Moderate	2
**Aspirin**	Valsartan, Telmisartan	N02	C09	PD-Renal function (elderly)	Moderate	7
	Ramipril				Moderate	3
**Azithromycin**	Mirtazapine	J01	N06	PD-QT prolongation	Monitor	2
**Carvedilol**	Dabigatran	C07	B01	PK-P-gp inhibition	Monitor	2
**Clopidogrel**	Esomeprazole, Omeprazole	B01	A02	PK-CYP inhibition	SUA	10
**Dexamethasone**	Levofloxacin, Ciprofloxacin	H02	J01	PD-Risk of tendon rupture	Moderate	16
**Digoxin**	Esomeprazole	C01	A02	PK-P-gp inhibition	Monitor	2
	**Azithromycin**	C01	J01		Monitor	2
**Diltiazem**	Rivaroxaban	C08	B01	PK-CYP, P-gp inhibition	Monitor	2
**Enoxaparin**	Dabigatran	B01	B01	PD-INR-monitor	SUA	2
	**Dexamethasone,** **Methylprednisolone**		H02		Moderate	91
	Budesonide		R03		Moderate	28
	**Azithromycin** Piperacilin Ceftaroline		J01		Moderate	21
					Moderate	12
					Moderate	6
	Citalopram		C09		Moderate	4
	Irbesartan, Telmisartan		C09	PD-hyperkalemia	Moderate	6
	Ramiprin		N06		Moderate	5
**Escitalopram**	Esomeprazole	N06	A02	PK-CYP inhibition	Monitor	7
	Leuprolide		L02	QT prolongation	SUA	3
**Gliclazide**	Furosemide	A10	C03	PD-hyperglycemia	Moderate	2
	Aspirin		N02	PD-hypoglycemia	Moderate	2
**Haloperidol**	Quetiapine	N05	N05	PD-QT prolongation	Monitor	3
**Indacaterol**	Formoterol	R03	C07	PD-acute bronchospasm	Moderate	2
**Insulin**	Levofloxacin	A10	J01	PD-blood glucose	Monitor	2
**Ipratropium**	Quetiapine	R03	N05	PD-hypoglycemia	Monitor	7
**Methylprednisolone**	Levofloxacin	H02	J01	PD-risk of tendon rupture	Moderate	5
**Quetiapine**	Ciprofloxacin, Levofloxacin	N05	J01	PD-QT prolongation	Monitor	5
	Sertraline		N06		Monitor	2
	Risperidone		N05		Monitor	2
**Ramipril**	Metformin	C09	A10	PD-hypoglycemia	Moderate	3
**Salbutamol**	Levofloxacin	R03	J01	PD-QT prolongation	Monitor	6
	Quetiapine		N05		Monitor	3
	Escitalopram Fluoxetine		N06		Monitor	2
					Monitor	2
	Bisoprolol		C07	PD-acute bronchospasm	Moderate	3
**Spironlactone**	KCl	C03	A12	PD-hyperkalemia	Monitor	2

PK: pharmacokinetic; PD: pharmacodynamic; DDI: drug-drug interactions; CYP: cytochrome P450; Ct: plasma concentration, QT: interval between the heart’s contraction and relaxation.

## Data Availability

During the data collection and analysis, all procedures were followed to ensure the confidentiality of the participants in accordance with EU directives and the General Data Protection Regulation (GDPR). The data presented in the study are available for use upon reasonable request/permission from the corresponding author. The data are not publicly available due to privacy statements and ethical reasons that were included in the informed consent form signed by the participants.

## Supplementary Materials

The following supporting information can be downloaded at: https://www.mdpi.com/article/10.3390/jcm11237172/s1, Figure S1: Clinical values of COVID-19 hospitalized patients; Table S1: ST 1 Drug combinations that may lead in pharmacokinetic DDIs as they recorded in the current study. Table S2: Drug combinations that may lead in pharmacodynamic DDIs as they recorded in the current study.

## Appendix A

Drugs were classified and presented in figures and tables according to the Anatomical Therapeutic Chemical (ATC) classification system. The table in the appendix shows the pharmacological subgroups of drugs that were classified into the respective ATC groups.

**Table A1:** ATC classification (2^nd^ level, anatomical group-therapeutic subgroup) of drugs in the study.

ATC-Codes	Drug Category	Pharmacological Subgroup
A02	Drugs for acid-related disorders	Proton pump inhibitors
A10	Drugs used in diabetes	Biguanides; dipeptidyl peptidase 4 (DPP-4) inhibitors; sodium-glucose co-transporter 2 (SGLT2) inhibitors
A11	Vitamins	Vitamin B-complex
A12	Mineral supplements	Calcium, combinations with vitamin D; potassium; magnesium
B01	Antithrombotic agents	Vitamin-K antagonists; heparin group; direct oral anticoagulants
B03	Antianemic preparations	Ferrous supplements
C01	Cardiac therapy	Cardiac glycosides; antiarrhythmics; Vasodilators
C02	Antihypertensives	Imidazoline receptor agonists
C03	Diuretics	Thiazides; sulfonamides; aldosterone antagonists
C07	β-blockers	selective β-blockers, α-and β-blockers
C08	Ca^2+^ channel blockers (CCBs)	CCBs with vascular effects; CCBs with direct cardiac effects
C09	Agents acting on the renin–angiotensin system	ACE inhibitors; ARBs
C10	Lipid modifying agents	HMG CoA reductase inhibitors (statins)
G03	Sex hormones and modulators of the genital system	Progestogens and estrogens
G04	Urologicals	Drugs used in benign prostatic hypertrophy
H02	Corticosteroids for systemic use	Glucocorticoids
H03	Thyroid therapy	Thyroid hormones
J01	Antibacterials for systemic use	Penicillins; macrolides; quinolones
J02	Antimycotics for systemic use	Imidazole and triazole derivatives
J05	Antivirals for systemic use	Direct acting antiviral drugs (remdesivir)
L01	Antineoplastic agents	Protein kinase inhibitors
L02	Endocrine therapy	Gonadotropin-releasing hormone analogs
L04	Immunosuppressants	Interleukin inhibitors; antimetabolites
M04	Antigout preparations	Uric acid inhibitors
N02	Analgesics	Analgesics and antipyretics
N03	Antiepileptics	Carboxamide (carbamazepine) and fatty acid (valproic acid) derivatives
N04	Anti-Parkinson drugs	Dopaminergic agents
N05	Psycholeptics	Antipsychotics, anxiolytics, and sedatives
N06	Psychoanaleptics	Antidepressants
R03	Drugs for obstructive airway diseases	β-2-receptor agonists; glucocorticoids
