# Supplementary material for: Drug-Drug Interactions among Patients Hospitalized with COVID-19 in Greece
Source: J Clin Med. 2022 Dec 2;11(23):7172. doi: 10.3390/jcm11237172 (PMC9740400; doi:10.3390/jcm11237172)
Supplement: Supplementary file 1 [file jcm-11-07172-s001.zip › jcm-1997879-supplementary.pdf]

Supplementary Material: Drug-drug interactions among patients hospitalized with COVID-19 in Greece

Marios Spanakis [1,2*], Petros Ioannou[3], Sotiris Tzalis[3], Vasiliki Papakosta[3], Evridiki Patelarou[1], Nikos Tzanakis[4], Athina Patelarou[1] and Diamantis P. Kofteridis[3]

1 Department of Nursing, School of Health Sciences, Hellenic Mediterranean University, 71004 Heraklion, Greece
2 Computational Biomedicine Laboratory, Institute of Computer Science, Foundation for Research and Technology-Hellas (FORTH), 70013 Heraklion, Greece
3 Department of Internal Medicine and Infectious Diseases, University Hospital of Heraklion, 71110 Heraklion, Greece
4 Department of Respiratory Medicine, University Hospital of Heraklion, Medical School, University of Crete, 7010013 Heraklion, Greece
* Correspondence: marspan@ics.forth.gr

The supplementary file contains additional information as supplementary figures (SF) and/or tables (ST) regarding the scientific data that were presented in the manuscript

SF1. Clinical values of COVID-19 hospitalized patients. Dashed lines show the normal range

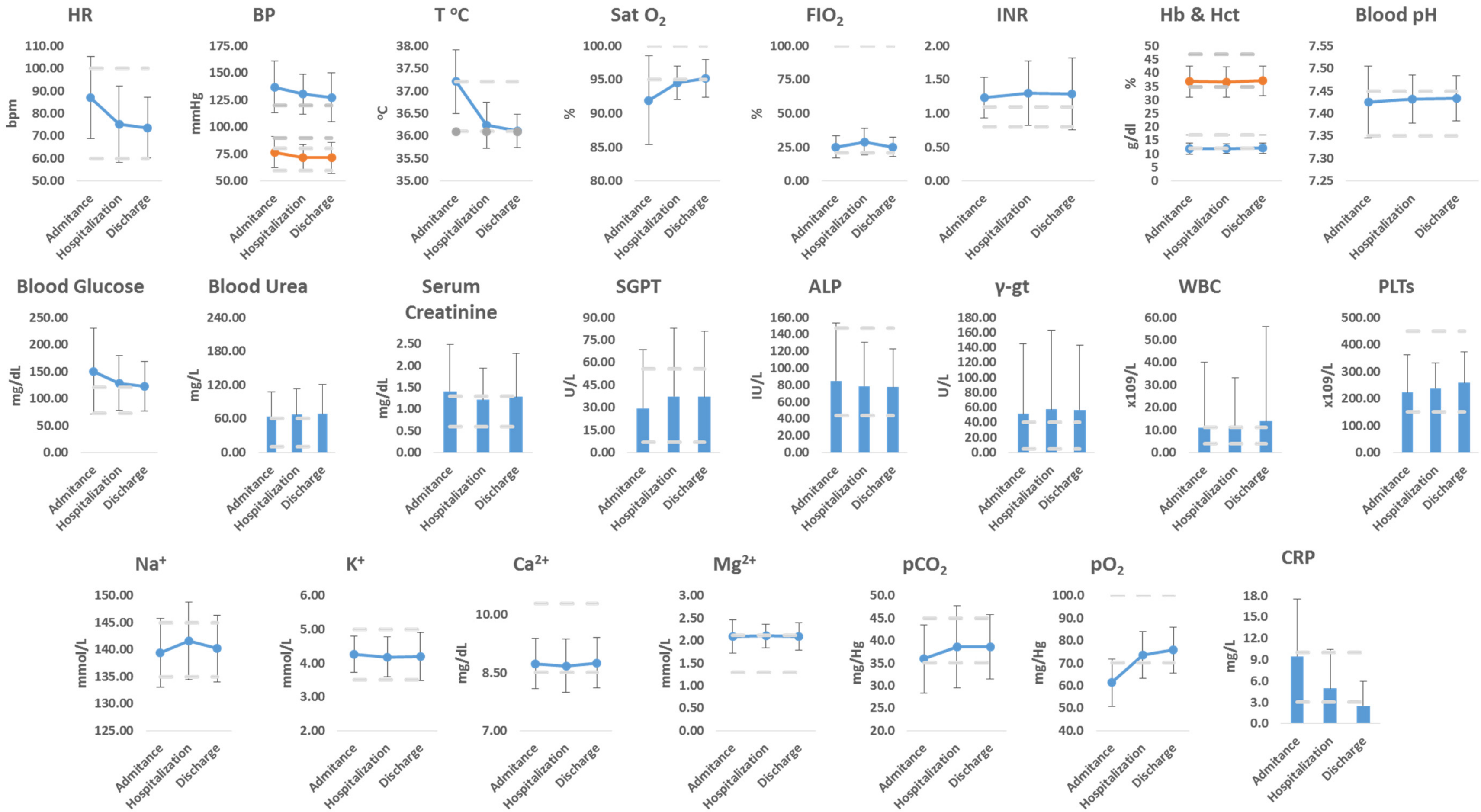

(HR: Heart Rate, BP: blood pressure; T: Temperature; Sat O2: Saturated O2; FIO2: fraction of inspired oxygen; INR: international normalised ratio; Hb: Hemoglobin; Hct: Hematocrit; SGPT: serum glutamic-pyruvic transaminase; ALP: Alkaline phosphatase; γ-gt: gamma-glutamyl transferase; WBC: white blood cells; PLTs: platelets; PCO2: partial pressure of carbon dioxide; pO2: partial pressure of oxygen; CRP: C-reactive protein)

ST 1 Drug combinations that may lead in pharmacokinetic DDIs as they recorded in the current study. SUA: Serious-Use alternative, Monitor: use with caution-monitor, Moderate: moderate-minor, occurrence: number of cases

# DDIs	Drug A	**Drug B**	Occurrence	**Significance**	**Pharmacological mechanism**
1	Acenocoumarol	Esomeprazole	3	Moderate	Inhibition of CYP mediated metabolism
2	Acenocoumarol	Allopurinol	1	Moderate	Modulation of anticoagulation action and altered INR-monitor
3	Acenocoumarol	Remdesivir	3	Monitor	Modulation of anticoagulation action and altered INR-monitor
4	Allopurinol	Furosemide	11	Monitor	Increased serum urate & Ct of metabolite
5	Amiodarone	Atorvastatin	2	Moderate	P-glycoprotein (MDR1) efflux transporter inhibition-increased bioavailability
6	Amiodarone	Silodosin	1	Monitor	P-glycoprotein (MDR1) efflux transporter inhibition-increased bioavailability
7	Amiodarone	Rivaroxaban	1	Monitor	Inhibition of CYP mediated metabolism
8	Amiodarone	Metformin	2	Moderate	Renal tubular clearance
9	Amiodarone	Metoprolol	1	Monitor	Inhibition of CYP mediated metabolism
10	Amiodarone	Dabigatran	1	Monitor	P-glycoprotein (MDR1) efflux transporter inhibition-increased bioavailability
11	Amiodarone	Tamsulosin	1	Moderate	Inhibition of CYP mediated metabolism
12	Amlodipine	Tacrolimus	1	Monitor	Inhibition of CYP mediated metabolism & P-gp transport
13	Amlodipine	Atorvastatin	1	Moderate	Inhibition of CYP mediated metabolism & P-gp transport
14	Anakinra	Alprazolam	1	Moderate	Restore 3uppressed CYP expression from inflamation
15	Apixaban	Amiodarone	1	Monitor	Inhibition of CYP mediated metabolism & P-gp transport
16	Apixaban	Dexamethasone	1	Monitor	Induction of CYP mediated metabolism
17	Azithromycin	Simvastatin	1	Moderate	Inhibition of CYP mediated metabolism
18	Bosutinib	Esomeprazole	1	Monitor	Reduced bioavailability due to pH dependent solubility
19	Bupropion	Clopidogrel	1	Monitor	Inhibition of CYP mediated metabolism
20	Bupropion	Metformin	1	Moderate	Inhibition of OCT2 mediated transport
21	Bupropion	Tamsulosin	1	Moderate	Inhibition of CYP mediated metabolism
22	Carbamazepine	Risperidone	1	Moderate	Induction of CYP mediated metabolism
23	Carbamazepine	Olanzapine	1	Moderate	Induction of CYP mediated metabolism
24	Carbamazepine	Esomeprazole	1	Moderate	Induction of CYP mediated metabolism
25	Carbamazepine	Dexamethasone	1	Moderate	Induction of CYP mediated metabolism
26	Carvedilol	Dabigatran	2	Monitor	P-glycoprotein (MDR1) efflux transporter inhibition-increased bioavailability
27	Carvedilol	Paroxetine	1	Monitor	Inhibition of CYP mediated metabolism
28	Carvedilol	Calcium	1	Moderate	Ca2+ reduce the bioavailability of β-blockers
29	Clopidogrel	Esomeprazole	**9**	SUA	Inhibition of CYP mediated metabolism
30	Clopidogrel	Lansoprazole	1	Monitor	Inhibition of CYP mediated metabolism

31	Dexamethasone	Solifenacin	2	Moderate	Induction of CYP mediated metabolism
32	Dexamethasone	Propafenone	1	Moderate	Induction of CYP mediated metabolism
33	Dexamethasone	Tacrolimus	1	Monitor	Induction of CYP mediated metabolism and P-gp transport
34	Digoxin	Alprazolam	1	Monitor	Protein binding competition
35	Digoxin	Esomeprazole	**2**	Monitor	P-glycoprotein (MDR1) efflux transporter inhibition-increased bioavailability
36	Digoxin	Azithromycin	2	Monitor	P-glycoprotein (MDR1) efflux transporter inhibition-increased bioavailability
37	Digoxin	Spironolactone	1	Moderate	Decrease tubular secretion
38	Diltiazem	Rivaroxaban	2	Monitor	Inhibition of CYP mediated metabolism & P-gp transport
39	Diltiazem	Atorvastatin	1	Monitor	Inhibition of CYP mediated metabolism & P-gp transport
40	Diltiazem	Amlodipine	1	SUA	Inhibition of CYP mediated metabolism
41	Diltiazem	Eplerenone	1	SUA	Inhibition of CYP mediated metabolism & P-gp transport
42	Diltiazem	Proglitazone	1	Monitor	Inhibition of CYP mediated metabolism
43	Diltiazem	Tamsulosin	1	Monitor	Inhibition of CYP mediated metabolism
44	Enoxaparin	Azithromycin	**21**	Moderate	Reduced metabolism
45	Esomeprazole	Escitalopram	7	Monitor	Inhibition of CYP mediated metabolism
46	Esomeprazole	Tacrolimus	1	Monitor	Inhibition of CYP mediated metabolism
47	Ferrous gluconate	Esomeprazole	2	Moderate	Reduced bioavailability due to pH dependent solubility
48	Ferrous gluconate	Omeprazole	1	Moderate	Reduced bioavailability due to pH dependent solubility
49	Fluconazole	Esomeprazole	1	Moderate	Inhibition of CYP mediated metabolism
50	Fluconazole	Escitalopram	1	Monitor	Inhibition of CYP mediated metabolism
51	Fluconazole	Amlodipine	1	Monitor	Inhibition of CYP mediated metabolism
52	Fluconazole	Tacrolimus	1	SUA	Inhibition of CYP mediated metabolism
53	Isavuconazonium	Tacrolimus	1	Monitor	Inhibition of CYP mediated metabolism & P-gp transport
54	Isavuconazonium	Dexamethasone	1	Moderate	Induction of CYP mediated metabolism and P-gp transport
55	Levothyroxine	Esomeprazole	12	Moderate	Reduced bioavailability due to pH dependent solubility
56	Levothyroxine	Omeprazole	2	Moderate	Reduced bioavailability due to pH dependent solubility
57	Levothyroxine	Lansoprazole	2	Moderate	Reduced bioavailability due to pH dependent solubility
58	Levothyroxine	Calcium	2	Moderate	Inhibition of GI absorption reduced bioavailability
59	Levothyroxine	Rabeprazole	1	Moderate	Reduced bioavailability due to pH dependent solubility
60	Magnesium	Levofloxacin	1	Moderate	Modulation of GI absorption
61	Methotrexate	Gliclazide	1	Moderate	Protein binding competition
62	Methylprednisolone	Silodosin	1	Moderate	Induction of CYP mediated metabolism
63	Nifedipine	Atorvastatin	1	Monitor	Inhibition of CYP mediated metabolism & P-gp transport
64	Omeprazole	Ciprofloxacin	1	Moderate	Reduced bioavailability due to pH dependent solubility

65	Omeprazole	Olanzapine	1	Moderate	Induction of CYP mediated metabolism
66	Omeprazole	Diazepam	1	Moderate	Inhibition of CYP mediated metabolism
67	Omeprazole	Clopidogrel	1	SUA	Inhibition of CYP mediated metabolism
68	Omeprazole	Duloxetine	1	Monitor	Reduced bioavailability due to pH dependent solubility
69	Proglitazone	Rosuvastatin	1	Moderate	Inhibition of OATP1B1 mediated transport-risk of myopathy
70	Ranolazine	Rosuvastatin	1	Moderate	Inhibition of OATP1B1 mediated transport-risk of myopathy
71	Sertraline	Haloperidol	1	Monitor	Inhibition of CYP mediated metabolism
72	Tamsulosin	Fluoxetine	1	Monitor	Inhibition of CYP mediated metabolism

ST 2 Drug combinations that may lead in pharmacodynamic DDIs as they recorded in the current study. SUA: Serious-Use alternative, Monitor: use with caution-monitor, Moderate: moderate-minor, occurrence: number of cases

DDIs #	Drug A	Drug B	Occurrence	Significance	Description
**1**	Abatacept	Adalimumab	1	SUA	PD-synergism increased risk for serious infection
**2**	Acenocoumarol	Rosuvastatin	2	Moderate	Modulation of anticoagulation action and altered INR-monitor
**3**	Acenocoumarol	Methylprednisolone	3	Monitor	Modulation of anticoagulation action and altered INR-monitor
**4**	Acenocoumarol	Piperacilin	1	Moderate	Modulation of anticoagulation action and altered INR-monitor
**5**	Acenocoumarol	Ceftriaxone	2	SUA	Modulation of anticoagulation action and altered INR-monitor
**6**	Acenocoumarol	Dexamethasone	1	Moderate	Modulation of anticoagulation action and altered INR-monitor
**7**	Alprazolam	Moxonidine	1	Moderate	PD synergism, sedation
**8**	Amiodarone	Citalopram	1	Monitor	QT prolongation
**9**	Amiodarone	Formoterol	1	Monitor	QT prolongation
**10**	Amiodarone	Ciprofloxacin	1	Monitor	QT prolongation
**11**	Amiodarone	Quetiapine	1	Monitor	QT prolongation
**12**	Amiodarone	Escitalopram	1	Monitor	QT prolongation
**13**	Amiodarone	Levofloxacin	1	Monitor	QT prolongation
**14**	Amisulpride	Haloperidol	1	Monitor	QT prolongation
**15**	Amisulpride	Quetiapine	1	Monitor	QT prolongation
**16**	amitriptyline	Risperidone	1	Monitor	QT prolongation
**17**	Amlodipine	Calcium	1	Moderate	PD-antagonism of Ca2+ with Ca2+ channel blockers
**18**	Apixaban	Escitalopram	1	Moderate	SSRIs may alter serotonin release from platelets and induce bleeding
**19**	Aspirin	Olmesartan	2	Moderate	Kidney function, NSAIDs & ARBs may produce deterioration in renal function (elderly)

**20**	Aspirin	Telmisartan	3	Moderate	Kidney function, NSAIDs & ARBs may produce deterioration in renal function (elderly)
**21**	Aspirin	Duloxetine	1	Monitor	SSRIs may alter serotonin release from platelets and induce bleeding
**22**	Aspirin	Valsartan	4	Moderate	Kidney function, NSAIDs & ARBs may produce deterioration in renal function (elderly)
**23**	Aspirin	Carvedilol	5	Moderate	PD antagonism
**24**	Aspirin	Citalopram	2	Moderate	SSRIs may alter serotonin release from platelets and induce bleeding
**25**	Aspirin	Escitalopram	2	Moderate	SSRIs may alter serotonin release from platelets and induce bleeding
**26**	Aspirin	Irbesartan	1	Moderate	Kidney function, NSAIDs & ARBs may produce deterioration in renal function (elderly)
**27**	Aspirin	Ramipril	3	Moderate	Reduce renal function & antihypertensive effect of ACE inhibitors
**28**	Aspirin	Candesartan	1	Moderate	Kidney function, NSAIDs & ARBs may produce deterioration in renal function (elderly)
**29**	Aspirin	Vortioxetine	1	Moderate	SSRIs may alter serotonin release from platelets and induce bleeding
**30**	Aspirin	Metoprolol	1	Moderate	PD antagonism
**31**	Aspirin	Bisoprolol	1	Moderate	PD antagonism
**32**	Aspirin	Perindopril	1	Moderate	Reduce renal function & antihypertensive effect of ACE inhibitors
**33**	Atenolol	Alprazolam	1	Moderate	Addititve hypotensive effects
**34**	Atenolol	Zolpidem	1	Moderate	Addititve hypotensive effects
**35**	Atenolol	Insulin	1	Moderate	Hypoglycemia
**36**	Azithromycin	Mirtazapine	2	Monitor	QT prolongation
**37**	Biperiden	Levomepromazine	1	Moderate	CNS/Respiratory depression
**38**	Bisoprolol	Silodosin	1	Moderate	PD synergism
**39**	Bisoprolol	Formoterol	1	Moderate	PD antagonism
**40**	Carbamazepine	Levomepromazine	1	Moderate	PD synergism, sedation
**41**	Carbamazepine	Biperiden	1	Moderate	CNS/Respiratory depression
**42**	Carvedilol	Salbutamol	1	Moderate	PD antagonism
**43**	Ciprofloxacin	Fluoxetine	1	Monitor	QT prolongation
**44**	Citalopram	Furosemide	2	Moderate	Risk for hypnatriemia
**45**	Citalopram	Clopidogrel	1	Moderate	SSRIs may alter serotonin release from platelets and induce bleeding
**46**	Dexamethasone	Aspirin	10	Moderate	GI Side effects
**47**	Dexamethasone	Levofloxacin	11	Moderate	Quinolones & corticosteroids increase risk of tendon rupture
**48**	Dexamethasone	Ciprofloxacin	5	Moderate	Quinolones & corticosteroids increase risk of tendon rupture
**49**	Donepezile	Paroxetine	1	Monitor	QT prolongation
**50**	Donepezile	Haloperidol	1	Moderate	Additive anticholinergic effects,

**51**	Donepezile	Olanzapine	1	Moderate	Additive anticholinergic effects,
**52**	Drospirenone	Spironolactone	1	Moderate	Risk for hyperkalemia
**53**	Drospirenone	Candesartan	1	Moderate	Risk for hyperkalemia
**54**	Drospirenone	Carvedilol	1	Moderate	Risk for hyperkalemia
**55**	Empagliflozin	Furosemide	1	Moderate	SGLT-2 inhibitors may potentiate the diuretic/ hypotensive effects of loop diuretics/ loop diuretics can cause hyperglycemia and may reduce the therapeutic effects of SGLT-2 inhibitors
**56**	Empagliflozin	Spironolactone	1	Moderate	SGLT-2 inhibitors may potentiate the diuretic/ hypotensive effects of loop diuretics/ loop diuretics can cause hyperglycemia and may reduce the therapeutic effects of SGLT-2 inhibitors
**57**	Enoxaparin	Methylprednisolone	**32**	Moderate	Corticosteroids may decrease anticoagulant activity/impair vascular integrity
**58**	Enoxaparin	Ceftaroline	6	Moderate	cephalosporins may decrease prothrombin activity
**59**	Enoxaparin	Budesonide	**28**	Moderate	Corticosteroids may decrease anticoagulant activity/impair vascular integrity
**60**	Enoxaparin	Dexamethasone	59	Moderate	Corticosteroids may decrease anticoagulant activity/impair vascular integrity
**61**	Enoxaparin	Ceftriaxone	**16**	Moderate	cephalosporins may decrease prothrombin activity
**62**	Enoxaparin	Piperacilin	**12**	Moderate	PD-synergism anticoagulation
**63**	Enoxaparin	Perindopril	**1**	Moderate	LMW-Heps may suppress adrenal aldosterone secretion. Hyperkalemia (also from ACE inhb)
**64**	Enoxaparin	Irbesartan	7	Moderate	LMW-Heps may suppress adrenal aldosterone secretion. Hyperkalemia (also from ACE inhb)
**65**	Enoxaparin	Ramipril	**5**	Moderate	LMW-Heps may suppress adrenal aldosterone secretion. Hyperkalemia (also from ACE inhb)
**66**	Enoxaparin	Dabigatran	2	SUA	PD synergism enhanced bleeding risk
**67**	Enoxaparin	Citalopram	4	Moderate	PD synergism enhanced bleeding risk
**68**	Enoxaparin	Telmisartan	**2**	Moderate	LMW-Heps may suppress adrenal aldosterone secretion. Hyperkalemia (also from ACE inhb)
**69**	Enoxaparin	Prednisolone	1	Moderate	Corticosteroids may decrease anticoagulant activity/impair vascular integrity
**70**	Enoxaparin	Valsartan	1	Moderate	LMW-Heps may suppress adrenal aldosterone secretion. Hyperkalemia (also from ACE inhb)
**71**	Eplerenone	Potassium Chloride	1	Monitor	Risk for hyperkalemia
**72**	Escitalopram	Levofloxacin	1	Monitor	QT prolongation
**73**	Escitalopram	Leuprolide	3	SUA	QT prolongation
**74**	Escitalopram	Clopidogrel	2	Moderate	SSRIs may alter serotonin release from platelets and induce bleeding
**75**	Escitalopram	Mirtazapine	1	Monitor	Risk for serotonine syndrome
**76**	Furosemide	Sertraline	1	Moderate	PD-synergism hyponatriemia

**77**	Furosemide	Metformin	1	Moderate	Hypoglycemia
**78**	Gliclazide	Furosemide	2	Moderate	PD antagonism
**79**	Gliclazide	Aspirin	2	Moderate	Hypoglycemia
**80**	Gliclazide	Lisinopril	1	Moderate	Hypoglycemia
**81**	Haloperidol	Quetiapine	3	Monitor	QT prolongation
**82**	Indacaterol	Propafenone	1	Monitor	QT prolongation
**83**	Indacaterol	Quetiapine	1	Monitor	QT prolongation
**84**	Indacaterol	Furosemide	1	Moderate	Risk of hypokalemia
**85**	Indacaterol	Bisoprolol	2	Moderate	Antagonism -brochospasm
**86**	Insulin	Risperidone	1	Moderate	Antipsychotics can produce hyperglycemia and alter blood glucose control
**87**	Insulin	Levofloxacin	2	Monitor	Quinolone antibiotic administration may result in hyper- or hypoglycemia
**88**	Ipratropium	Haloperidol	**1**	Moderate	Additive anticholinergic effects,
**89**	Ipratropium	Quetiapine	7	Monitor	Hypoglycemia
**90**	Leuprolide	Escitalopram	1	Monitor	QT prolongation
**91**	Levodopa	Haloperidol	1	SUA	PD-antagonism decreased effect of levodopa
**92**	Levodopa	Quetiapine	1	Monitor	PD-antagonism decreased effect of levodopa
**93**	Levodopa	Telmisartan	1	Moderate	PD-synergism hypotension
**94**	Levofloxacin	Fluoxetine	1	Monitor	QT prolongation
**95**	Linezolid	Salbutamol	1	Monitor	MAOIs can potentiate the cardiovascular ADE of β-2-adrenergic agonists
**96**	Linezolid	Escitalopram	1	SUA	Risk for serotonine syndrome
**97**	Linezolid	Aripiprazole	1	Monitor	Risk for serotonine syndrome
**98**	Methylprednisolone	Aspirin	6	Moderate	GI Side effects
**99**	Methylprednisolone	Metformin	1	Moderate	PD antagonism
**100**	Methylprednisolone	Levofloxacin	5	Moderate	Quinolones & corticosteroids increase risk of tendon rupture
**101**	Methylprednisolone	Moxifloxacin	**1**	Moderate	Quinolones & corticosteroids increase risk of tendon rupture
**102**	Methylprednisolone	Ciprofloxacin	2	Moderate	Quinolones & corticosteroids increase risk of tendon rupture
**103**	Olanzapine	Levomepromazine	1	Moderate	PD-synergism & excessive parasympatholytic effects
**104**	Olanzapine	Codeine	1	Monitor	PD synergism, sedation
**105**	Olanzapine	Aclidinium	1	Moderate	Hypoglycemia
**106**	Olanzapine	Ipratropium	1	Moderate	Hypoglycemia

**107**	Olanzapine	Mirtazapine	1	Monitor	QT prolongation
**108**	Olanzapine	Chloropromazine	1	Monitor	QT prolongation
**109**	Paroxetine	Clopidogrel	1	Monitor	SSRIs may alter serotonin release from platelets and induce bleeding
**110**	Perindopril	Alogliptin	1	Moderate	Hypoglycemia
**111**	perphenazine	Risperidone	1	Monitor	QT prolongation
**112**	Prednisolone	Furosemide	2	Moderate	PD synergism risk for hypokalemia
**113**	Pregabalin	Sertraline	1	Monitor	Respiratory depression
**114**	Quetiapine	Propafenone	1	Monitor	QT prolongation
**115**	Quetiapine	Ciprofloxacin	3	Monitor	QT prolongation
**116**	Quetiapine	Codeine	1	Monitor	PD synergism, sedation
**117**	Quetiapine	Sertraline	2	Monitor	QT prolongation
**118**	Quetiapine	Risperidone	2	Monitor	QT prolongation
**119**	Quetiapine	Diazepam	1	Monitor	PD synergism, sedation
**120**	Quetiapine	Levofloxacin	2	Monitor	QT prolongation
**121**	Quetiapine	Fluoxetine	1	Monitor	QT prolongation
**122**	Ramipril	Potassium Chloride	1	Monitor	Risk for hyperkalemia
**123**	Ramipril	Metformin	3	Moderate	Hypoglycemia
**124**	Risperidone	Paroxetine	1	Monitor	QT prolongation
**125**	Rivaroxaban	Escitalopram	1	Moderate	SSRIs may alter serotonin release from platelets and induce bleeding
**126**	Salbutamol	Haloperidol	**1**	Monitor	QT prolongation
**127**	Salbutamol	Furosemide	3	Moderate	PD synergism risk for hypokalemia
**128**	Salbutamol	Propafenone	1	Monitor	QT prolongation
**129**	Salbutamol	Escitalopram	2	Monitor	QT prolongation
**130**	Salbutamol	Bisoprolol	3	Moderate	PD antagonism
**131**	Salbutamol	Levofloxacin	6	Monitor	QT prolongation
**132**	Salbutamol	Azithromycin	**1**	Monitor	QT prolongation
**133**	Salbutamol	Moxifloxacin	**1**	Monitor	QT prolongation
**134**	Salbutamol	Metoprolol	2	Moderate	PD antagonism
**135**	Salbutamol	Formoterol	1	Moderate	PD-synergism cardiovascular side effects
**136**	Salbutamol	Norfloxacin	1	Monitor	QT prolongation
**137**	Salbutamol	Fluoxetine	2	Monitor	QT prolongation
**138**	Salbutamol	Quetiapine	3	Monitor	QT prolongation
**139**	Salbutamol	Ciprofloxacin	1	Monitor	QT prolongation
**140**	Salmeterol	Bisoprolol	2	Moderate	PD antagonism

**141**	Salmeterol	Furosemide	1	Moderate	Risk of hypokalemia
**142**	Saxagliptin	Chloropromazine	1	Moderate	Antipsychotics can produce hyperglycemia and alter blood glucose control
**143**	Saxagliptin	Olanzapine	1	Moderate	Antipsychotics can produce hyperglycemia and alter blood glucose control
**144**	Sertraline	Clopidogrel	2	Moderate	SSRIs may alter serotonin release from platelets and induce bleeding
**145**	Sertraline	Olanzapine	1	Monitor	QT prolongation
**146**	Spironlactone	Candesartan	2	Moderate	Risk for hyperkalemia
**147**	Spironlactone	Potassium Chloride	2	Monitor	PD -synergism hyperkaliemia
**148**	Trazodone	Paroxetine	1	Monitor	QT prolongation
**149**	Vancomycin	Tacrolimus	1	Monitor	Risk for nephrotoxicity and/or ototoxicity.
**150**	Venlafaxine	Risperidone	1	Monitor	QT prolongation
**151**	Venlafaxine	Paroxetine	1	Monitor	Risk for serotonine syndrome
**152**	Venlafaxine	Mirtazapine	1	Monitor	QT prolongation
**153**	Venlafaxine	Azithromycin	1	Monitor	QT prolongation
**154**	Zolpidem	Alprazolam	1	Monitor	PD synergism, sedation
